# Metamorphosing Smiles Through Esthetic Vital Bleaching: A Twin Case Report

**DOI:** 10.7759/cureus.62748

**Published:** 2024-06-20

**Authors:** Swayangprabha Sarangi, Manoj Chandak, Aditya Patel, Mrinal Nadgouda, Palak Hirani, Kapil M Naladkar

**Affiliations:** 1 Conservative Dentistry and Endodontics, Sharad Pawar Dental College, Datta Meghe Institute of Higher Education and Research, Wardha, IND

**Keywords:** discoloration, mcinnes solution, hydrogen peroxide, vital bleaching, bleaching

## Abstract

Patients have been recently observed to have undergone a noticeable increase in the concern over tooth discoloration making it a common cosmetic issue. This cumulative number of visits to dental experts is in search of ways to whiten their teeth because they desire to improve their looks. Often, the desire for a brighter smile is associated with health and beauty perceptions. Thus, patients tend to go after what they think is the perfect form of an unflawed grin. Diverse treatment modalities exist for tooth discolorations, such as composite laminate and veneers, porcelain veneers, and partial crowns. Amongst these options, bleaching, therefore, appears to be a conservative manner of dealing with teeth discoloration. This twin case report shows that discoloration can be managed best through bleaching using McInnes solution, resulting in good outcomes. Using McInnes solution instead makes bleaching an affordable and conservative technique for removing stains.

## Introduction

The natural hue of permanent teeth typically ranges from grayish yellow to grayish white or yellowish white. Any deviations from this coloration may stem from either physiological or pathological factors, either originating within the body or induced externally [1]. Tooth color is predisposed by factors like transparency and thickness of enamel, color and density of underlying dentin, and hue of underlying pulp [2]. It serves as an indicator of oral health, habits, and age. Proper clinical assessment and history gathering are crucial for determining the cause and extent of tooth discoloration [1,2]. Discoloration can arise from surface stains or internal factors. The least invasive and conservative approach to treating such stains is tooth bleaching [3]. Tooth whitening has become a popular cosmetic dental procedure, with various methods available, including brushing, bleaching strips, pens, gels, and laser treatments [3]. The earliest tooth bleaching procedures were conducted in dental offices, with modern methods often utilizing high-concentration hydrogen peroxide, commonly known as "one-hour bleaching," ranging from 25% to 35% concentration [4,5].

As per the literature, the benefits of bleaching are numerous. Hence, this case report is presented to showcase the technique of vital tooth bleaching using hydrogen peroxide and the McInnes Solution. To ensure successful esthetic treatment for tooth discolorations, careful consideration must be given to selecting patients with conditions that are most likely to respond well to bleaching. Several factors significantly impact the outcome of bleaching treatment, including the concentration and duration of the bleaching agent's application, the nature of the tooth discoloration, the initial color of the teeth, and the age of the patient [5,6].

## Case presentation

Case 1

A 20-year-old female patient presented at the outpatient section of the department of Conservative Dentistry and Endodontics, Sharad Pawar Dental College, Wardha, expressing concerns about her discolored upper front teeth. During the examination, emphasis was placed on assessing key clinical parameters, including the patient's periodontal health, presence of gingival recession, and absence of decay. Additionally, inquiries were made regarding any history of tooth sensitivity. However, the patient in this case had no history of previous tooth sensitivity and decay. The patient's history revealed that she resided in an area with poor water quality. She also had a history of consumption of borewell water since childhood. She had no habit of consuming tobacco, areca nuts, and other related substances, which stated the discoloration to be purely endemic fluorosis (Grade 4, according to Dean's Fluorosis Index) in origin. 

Procedure of Treatment

**Figure 1:**
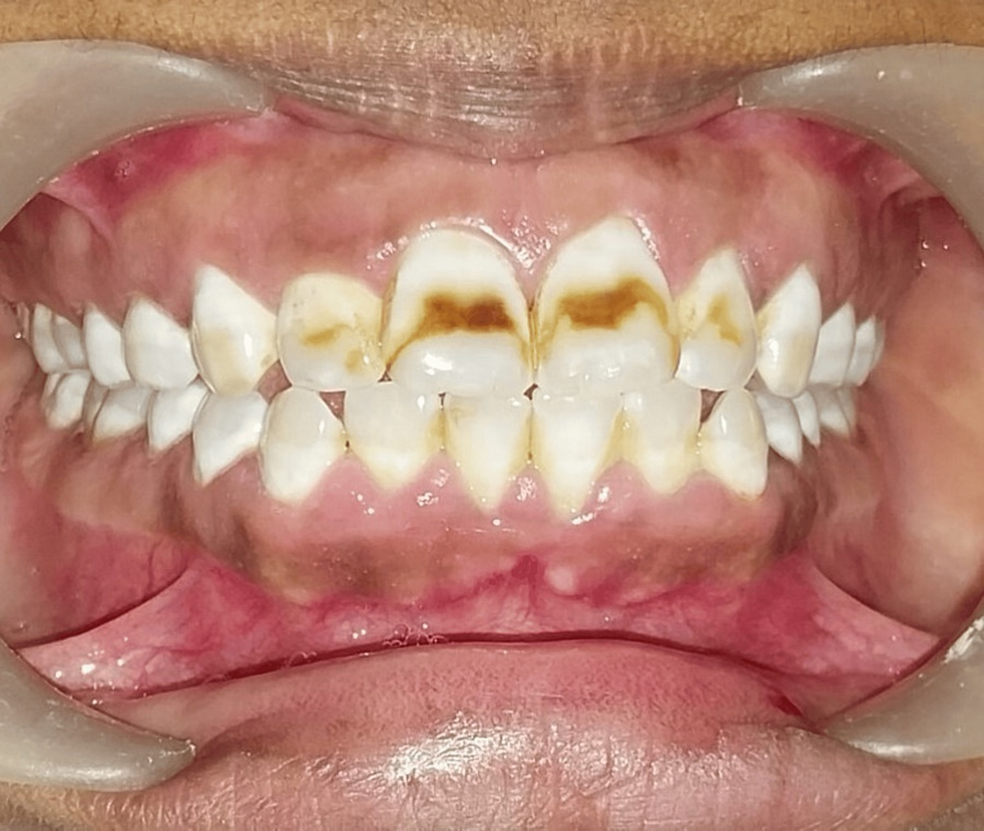
Intraoral preoperative picture showing moderate discoloration (Grade 4) in upper front teeth according to Dean's Fluorosis Index.

Intraoral pre-operative pictures were taken, and rubber dam application was performed (Figure 1).

**Figure 2:**
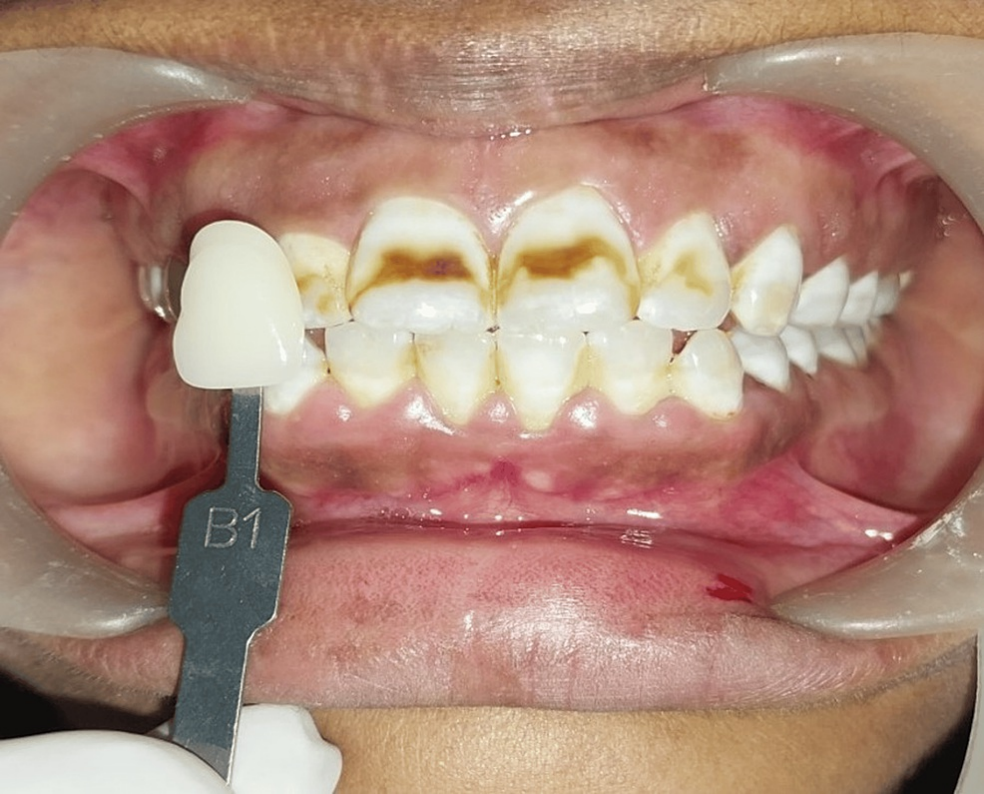
Shade verification using Vita classical shade guide.

The natural shade of the patient before treatment was B1, which was verified by a shade guide (Vita classical shade guide) (Figure 2).

**Figure 3:**
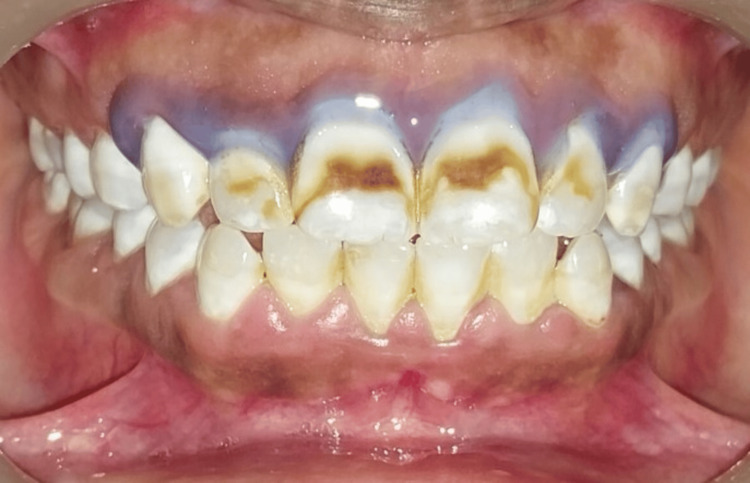
Application of gingival barrier in the upper front region of the maxillary arch.

Before bleaching treatment, the teeth underwent ultrasonic cleaning and drying, followed by the application of a gingival barrier (Pola Office, SDI Dental, Ireland) and light curing in a fanning motion (Figure 3).

**Figure 4:**
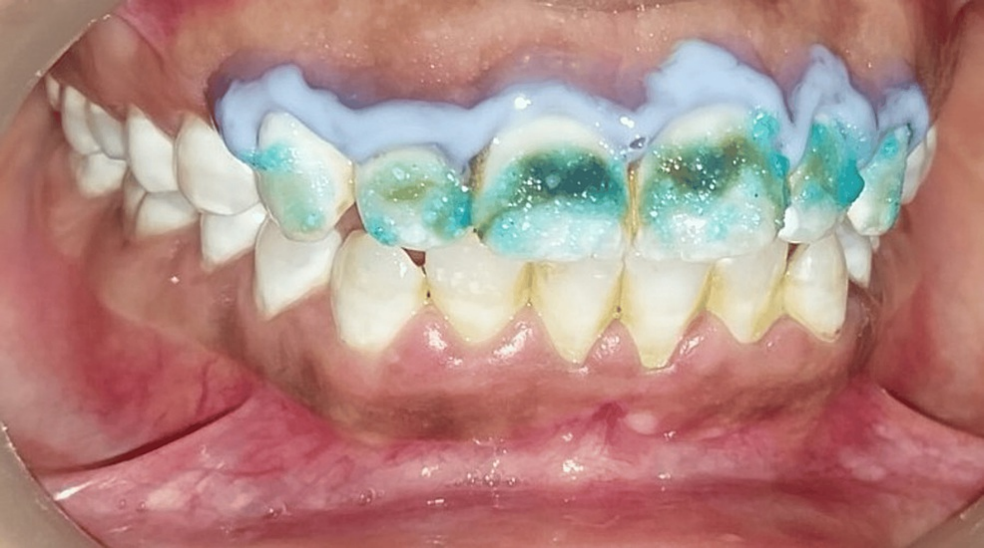
Bleaching agent (Pola Office, SDI Dental, Ireland) applied onto the facial surfaces.

A commercially available bleaching kit, Pola Office (SDI Dental Limited, Ireland), containing powder and hydrogen peroxide liquid was used. Both powder and liquid were assorted in a dispenser, maintaining a 1:1 ratio. The bleaching material, present in Pola Office containing 35% hydrogen peroxide, was utilized, enabling a rapid whitening process completed in under an hour. This formulation, together with potassium nitrate as a desensitizer, aims to minimize sensitivity, which is encountered in post-bleach procedures compared to alternative vital bleaching systems. The plunger of the hydrogen peroxide liquid syringe was carefully pulled back to release the pressure. Mixing was done using a brush tip applicator until the mixed gel was homogeneous. A dense layer of gel was then applied to all teeth, which showed the presence of staining (Figure 4).

Gel application time was eight minutes for all teeth as per the manufacturer's instructions, followed by using light for activation of the bleaching agent (Endoking, India). Intermittent suctioning was performed with the help of a surgical aspirator tip. A total of three applications were essential to complete the vital bleaching satisfactorily, after which the oral cavity was thoroughly rinsed with water. Once the procedure was finished, the gingival barrier was discarded. Further, the patient was asked about any sensitivity experienced post-treatment, to which she reported none. She was also instructed to avoid the consumption of tea, coffee, and colored food items for 2 weeks postoperatively.

**Figure 5:**
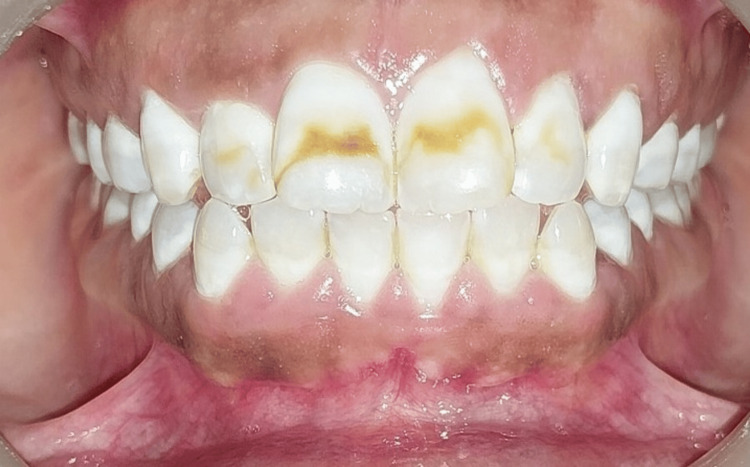
Postoperative picture following three visits of application of bleaching agent showing lightening of discolored teeth.

In this case, a bleaching light-emitting diode (LED) curing light was used, which is a cost-effective and easy-to-use light source for augmenting the in-office procedure. The patient was asked to return in 10 days to evaluate the results, on which the altered shade was matched as A3 (Figure 5).

Since the stains were very stubborn in nature and the superior esthetic demands of the patient, compelled to combine vital bleaching using McInnes solution (5 parts of 30% H_2_O_2_, 5 parts of 36% HCl and 1 part of anesthetic ether/alcohol) as another treatment method in this case. McInnes solution was freshly prepared, and ingredients were procured from the research laboratory in the institution and painted using a brush applicator tip. This solution was further activated with the help of a heated condenser (heated until red hot), which was placed perpendicular to the tooth surface for 5 seconds each 3 to 4 times. The procedure was repeated until the stains were lightened. The rubber dam was removed, and rinsing was done thoroughly using copious amounts of water.

**Figure 6:**
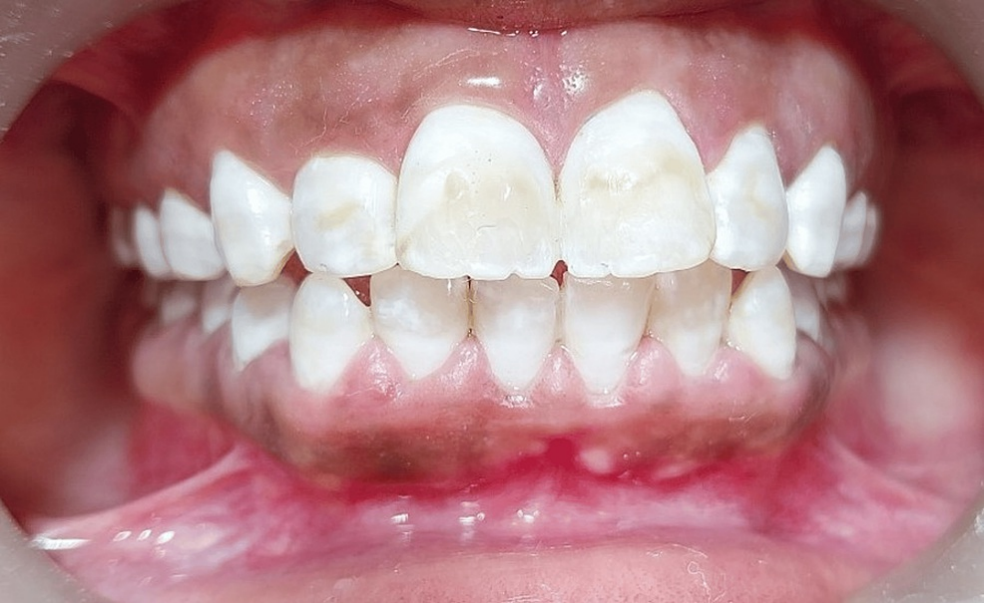
Final postoperative view following completion of bleaching using McInnes Solution.

A significant reduction in shade was observed post completion of the bleaching procedure, which was confirmed by standard classical VITA shade guide. Polishing of the teeth was done by prophylaxis paste ( Propol, DPI, India), which showed the desired shade improvement to A2 postoperatively. The patient noticed a marked improvement, had no postoperative sensitivity and was highly satisfied with the final result after treatment (Figure 6).

Case 2

**Figure 7:**
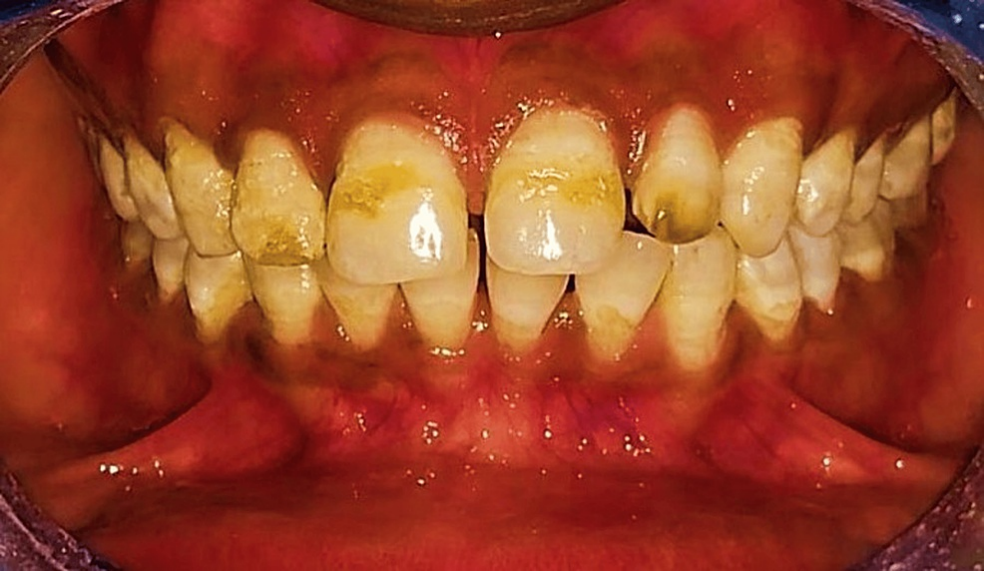
Preoperative intraoral picture showing Grade 4 fluorosis, i.e., a severe type according to Dean's Fluorosis Index. Note the pitting seen in the upper front teeth.

A 25-year-old male patient visited the department of Conservative Dentistry and Endodontics, Sharad Pawar Dental College, Wardha, citing tooth discoloration as his primary concern. Further inquiry revealed that he resided in an area with hard water quality, suggestive of a provisional diagnosis of endemic fluorosis. Clinical examination revealed moderate yellowish bands of discoloration on the buccal surfaces of his upper front teeth. Subsequently, the grade of fluorosis was Grade 4, i.e., a severe type of fluorosis for which bleaching was needed. The preoperative shade of the patient was recorded as B1 (Vita shade guide classic). He expressed a preference for in-office bleaching. Prior to the procedure, electric pulp testing was conducted on the maxillary anterior teeth, confirming their vitality. Thorough rinsing and drying of the teeth and gums were then performed (Figure 7).

**Figure 8:**
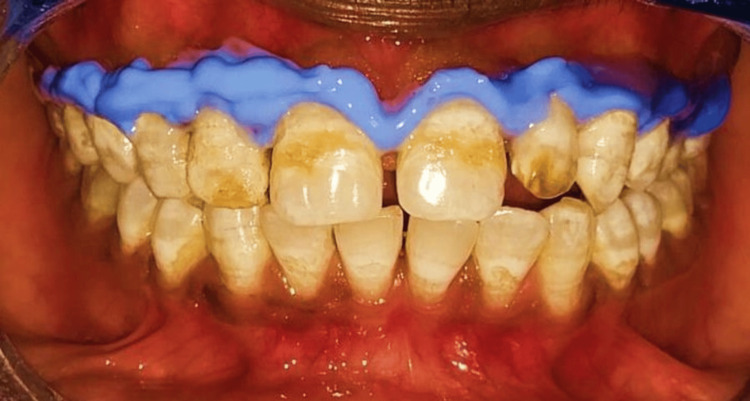
Application of gingival barrier prior to bleaching procedure.

Following the procedure as mentioned in the previous case, a gingival barrier (Pola Office, SDI Dental, Ireland) was dispensed along the gingival margins and cured using light activation device (Endoking, India) (Figure 8).

**Figure 9:**
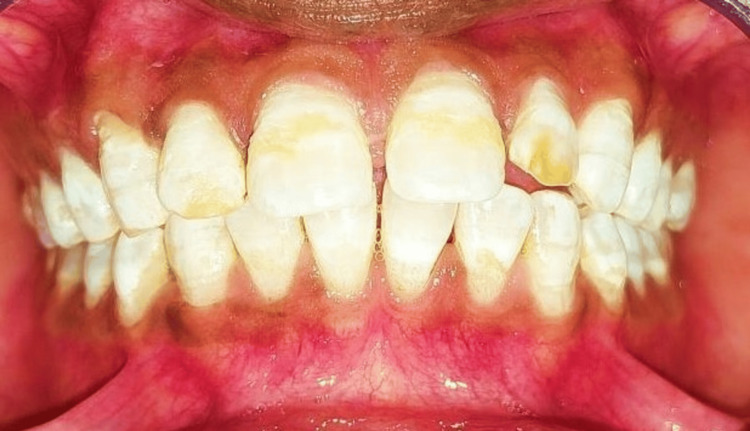
Intraoral picture following three visits of the bleaching procedure.

The Pola Office tooth whitening Kit (SDI Dental Limited, Ireland) was utilized for the procedure. The liquid containing hydrogen peroxide was mixed thoroughly with the clear powder by pressing the plunger of the syringe to form a paste to be painted onto the tooth surfaces. Saliva was suctioned periodically as required. Gel was kept on tooth surfaces for eight minutes according to the manufacturer's instructions and subsequently rinsed thoroughly from the tooth surfaces. The resin barrier was removed afterward. The patient underwent two follow-up appointments with a 10-day interval between each session, during which the procedure was repeated. The patient was instructed to return for an evaluation of the results (Figure 9).

**Figure 10:**
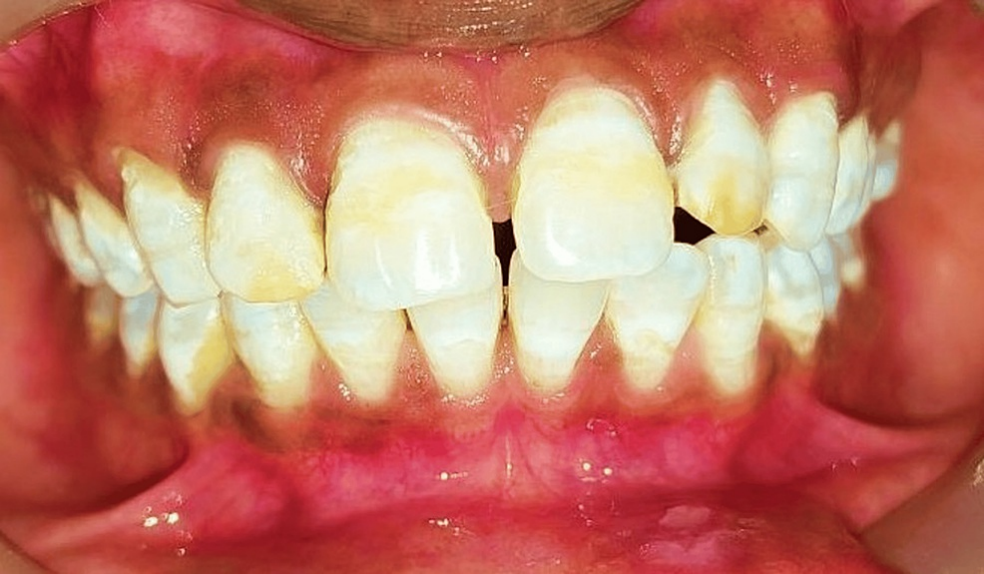
Final clinical postoperative view after completion of polishing and bleaching procedure

Using a standard visual examination, the shade change was evaluated, and the final shade was found to be A2. The tooth was polished using polishing paste (DPI Propol, India) and rubber polishing cups on a low-speed handpiece( Beijing Foshan, China) using 20,000 rpm for 2 mins to achieve smoothness of human surface as well as to remove abrasions and pitting. Noticeable shade change had occurred, and the patient was satisfied with the overall final shade. The patient was suggested to avoid the consumption of tea, coffee, and colored food for a period of two weeks postoperatively. He was also asked to report any sensitivity if experienced and undergo veneers for enhanced esthetics, but due to financial constraints, he denied further treatment (Figure 10).

## Discussion

The goal of the bleaching procedure is the restoration of the normal color of a tooth by decolorizing the stain with a powerful oxidizing bleaching agent [7]. Bleaching procedure can be indicated in almost all of the conditions where tooth discoloration occurs. However, its main contraindications are application in pregnant women, infants, and children under 10 years of age, patients who have teeth with exposed dentinal tubules and individuals who cannot quit smoking during the treatment period, and patients having severe sensitivity with the same teeth [8]. Regardless of the use of bleaching technique or product, the mechanism of action of bleaching agents is based on the release of active forms of oxygen as a function of the interaction of hydrogen peroxide with tooth structure [7].

Greater concentrations of hydrogen peroxide (25-40%) are used to accomplish faster tooth lightening. This overcomes the problems of patient compliance and manual dexterity and is also ideal for patients with high gag reflexes [8]. The potency of hydrogen peroxide peaks within the first 30 minutes of mixing, after which the free radicals are depleted. Depending on the manufacturer’s instructions, products are applied at a 2-3 mm thickness on the labial surfaces of the teeth [8,9]. Pola Office along with 35% hydrogen peroxide, also consists of potassium nitrate, which caused a reduction in postoperative sensitivity of the patient. Pulpal irritation and tooth sensitivity may be higher with the use of bleaching lights or heat application, and caution has been advised regarding their use in treatment [10,11].

 In Case 1, after this procedure, in-office vital bleaching was done using McInnes solution (1 part of 0.2% diethyl ether + 5 parts of 36% HCl + 5 parts of 30% hydrogen peroxide). McInnes solution was prepared just before the procedure to ensure its effectiveness, and the solution was applied to the teeth using brush applicator tips for 5-10 minutes at intervals [12]. McInnes solution offers several advantages, including cost-effectiveness, reduced chair-side time, and prompt treatment outcomes. By adequately isolating the treatment area with a rubber dam, this solution can be applied to either the entire dental arch or individual teeth [13, 14]. Dentists have the flexibility to adjust or halt the treatment as needed due to the easy control over the application of the solution. While the solution's acidic properties may lead to mild demineralization, this can be mitigated by prescribing desensitizing toothpaste, as implemented in our cases [15].

## Conclusions

Vital tooth bleaching is an effective and quicker modality of treatment that can considerably alter the exterior surfaces of teeth. For mild to moderate teeth discolorations in the office, vital teeth bleaching is a good and safe choice for dentists when used in the proper concentration of the agent. Additionally, stubborn stains can be effectively bleached using McInnes solution, keeping in mind its concentration and duration of application.
